# Regional associations between cerebrovascular disease and cholinergic white matter pathways in the Lewy body continuum

**DOI:** 10.1038/s41531-025-01118-5

**Published:** 2025-08-21

**Authors:** Anna Rennie, Milan Nemy, Cene Jerele, Iñigo Rodríguez-Baz, Victor Montal, Alexandre Bejanin, Milica G Kramberger, Dag Aarsland, Juan Fortea, Alberto Lleó, Eric Westman, Daniel Alcolea, Daniel Ferreira

**Affiliations:** 1 Division of Clinical Geriatrics, Center for Alzheimer Research, Department of Neurobiology, Care Sciences and Society, Karolinska Institute, Stockholm, Sweden; 2 Department of Biomedical Engineering and Assistive Technology, Czech Institute of Informatics, Robotics and Cybernetics, Czech Technical University in Prague, Prague, Czechia; 3 Clinical Institute of Radiology, University Medical Center Ljubljana, Ljubljana, Slovenia; 4 Hospital de la Santa Creu i Sant Pau, Biomedical Research Institute Sant Pau, Universitat Autònoma de Barcelona, Barcelona, Spain; 5 Center of Biomedical Investigation Network for Neurodegenerative Diseases (CIBERNED), Madrid, Spain; 6 Department of Neurology, University Medical Centre Ljubljana, Ljubljana, Slovenia; 7 Department of Old Age Psychiatry, Institute of Psychiatry, Psychology and Neuroscience, King’s College London, London, UK; 8 Centre for Age-Related Medicine, Stavanger University Hospital, Stavanger, Norway; 9 Facultad de Ciencias de la Salud, Universidad Fernando Pessoa Canarias, Las Palmas, España

**Keywords:** Neurodegeneration, Neurodegenerative diseases, Brain

## Abstract

Cerebrovascular disease is common in patients on the Lewy body (LB) continuum (dementia with Lewy bodies (DLB) and prodromal-DLB). White matter signal abnormality (WMSA) volume is higher in patients with LB than controls, both globally and in cholinergic white matter. However, it remains unknown if the higher WMSA in cholinergic white matter reflects selective cholinergic vulnerability or results from higher global WMSA. We modelled cingulate and external capsule cholinergic white matter pathways using MRI and segmented WMSA overlapping cholinergic pathways and per brain lobe. We found that patients on the LB-continuum (*n* = 33) had higher volume and proportion of WMSA in the cholinergic white matter compared to controls (*n* = 36), independent of global WMSA. Cholinergic WMSA was associated with neurodegeneration in the basal forebrain, decreased integrity of cingulate and external capsule pathways and attention and memory performance. These findings may suggest a selective vulnerability of cholinergic pathways in patients with LB.

## Introduction

Dementia with Lewy bodies (DLB) is a common type of dementia^1–3^. There are diagnostic criteria for both the dementia stage^4^ and the prodromal stage^5^. The diagnosis is currently based on clinical criteria as well as supportive or indicative biomarkers. Mild cognitive impairment with Lewy bodies (MCI-LB) is the most common prodromal form. Cholinergic dysfunction is well established in DLB via imaging studies^6–15^, postmortem studies^16^, and positive clinical response and decreased mortality when medicated with cholinesterase inhibitors^13,17,18^.

An important population of cholinergic neurons is located at the nucleus basalis of Meynert (NbM) in the basal forebrain. These neurons send widespread cholinergic white matter projections to the neocortex and hippocampus through medial and lateral pathways. The medial pathway projects from the NbM through the cingulum whilst the lateral pathway projects through the external capsule^19^. Cholinergic dysfunction in DLB is partly due to neurodegeneration of the NbM and nearby areas compared to controls^8,9^. There has recently been an interest in evaluating the cholinergic system non-invasively in vivo with an automated method based on magnetic resonance imaging (MRI). This method uses probabilistic tractography based on diffusion tensor imaging (DTI) and has been applied both in healthy^20,21^ and pathological^11,12,22–26^ ageing. Schumacher and colleagues^11^ and our group^22,25^ demonstrated that patients with DLB and Alzheimer’s Disease (AD) both at the dementia and MCI stages exhibit degeneration of these cholinergic pathways. Furthermore, Schumacher and colleagues^12^ additionally showed that degeneration of the pathway through the external capsule was associated with cognitive impairment in patients with DLB, AD, MCI-LB, and MCI-AD as well as with progression to dementia for the MCI patients, further highlighting the clinical impact of cholinergic dysfunction in DLB^11^.

While Lewy body pathology is the main driver of DLB, small vessel disease (SVD) is a common co-pathology in DLB^27,28^. Small vessel disease can be evaluated using MRI measures, for example, white matter signal abnormalities (WMSA) of presumed vascular origin^29^. The placement of WMSA may differ between DLB and other conditions^27^. Sarro et al.^30^ demonstrated that WMSA were more prevalent in the posterior white matter in DLB as compared with AD. We recently showed that patients with DLB and MCI-LB have a higher WMSA load in the cholinergic pathways compared to healthy controls, measured with a radiological visual rating scale^22^. Furthermore, Park et al.^6^ demonstrated that patients with DLB, AD or Parkinson’s disease with dementia (PDD) had more WMSA in cholinergic white matter areas compared to healthy controls, measured with a visual rating scale.

In our recent publication by Jerele and colleagues^22^, we observed that there were no associations of global measurements of WMSA with cognitive measures. Cognitive impairment in specific domains such as attention or episodic memory is correlated with alterations in specific brain networks and regions. Therefore, in the current study we hypothesise that using a newly developed regional approach that assesses cholinergic WMSA in different brain lobes and the insula can capture associations with cognitive measures, in contrast to the previous study reporting no associations with global WMSA^22^. Particularly, the insula is a region of early neurodegeneration in MCI-LB that continues to degenerate during manifest DLB and in relation with higher WMSA^31,32^. Moreover, it is still not known if the reported higher WMSA in cholinergic pathways is due to a globally higher WMSA volume or if it is due to a selective vulnerability of cholinergic pathways to SVD in DLB. Therefore, investigating cholinergic WMSA both in absolute measures and in proportion to the global or lobar WMSA may clarify whether the higher volume of cholinergic WMSA in DLB is a result of the overall higher global WMSA volume or it may reflect selective vulnerability of the cholinergic system to CVD. If the higher volume of cholinergic WMSA was a result of the overall higher global WMSA, there would be a difference in absolute measures but not in proportion of cholinergic WMSA between patients with DLB and controls. In contrast, a possible selective vulnerability could be reflected as significant group differences in both absolute and proportional measures.

The overall goal of this study was to investigate regional cholinergic WMSA placement within cholinergic white matter pathways along the Lewy body (LB) continuum. The LB continuum spans from MCI-LB to manifest DLB. Our first aim was to characterise the placement of WMSA and evaluate if LB patients are more likely to have WMSA in cholinergic pathways compared to controls. Our main measures of interest were the proportion of cholinergic WMSA, and we further report absolute measures of cholinergic WMSA for completeness of information and interpretation. Our second aim was to clarify the impact of WMSA on cholinergic integrity and their association with selected biomarkers of neurodegeneration such as volume of NbM and integrity of the cholinergic pathways measured through mean diffusivity (MD), as well as with AD co-pathology and measures of cognition.

## Results

### Cohort characteristics

Table 1 displays the cohort characteristics. The patients with LB group were statistically significantly older than the HC group and had fewer years of education. As expected, patients with LB had lower MMSE scores, after adjusting for age and education. There were no statistically significant differences in sex distribution between LB and HC.

**Table 1:** Cohort characteristics

	LB group	HC group	W-value/ χ²-value, p-value
*N*	33	36	
Age, years	76.0 (7)	66.5 (7)	W = 1006, *p* < 0.001
Sex, women *N* (%)	16 (48.5%)	21 (58.3%)	χ² (1, 69) = 0.334, *p* = 0.563
Education, years	11 (4)	16 (7.25)	W = 268, *p* < 0.001
Global cognition (MMSE, total score)	26.03 (4.5)	29.05 (1.8)	W = 135, *p* < 0.001
Attention (WAIS-III Digit span, total score)	12.21 (3.8)	13.75 (1.9)	W = 378, *p* = 0.03
Episodic memory (FCSRT free and cued recall, total score)	26.08 (22.5)	45.21 (4.7)	W = 121, *p* < .001
CSF Aβ 42/40 ratio	0.073 (0.03)	0.101 (0.03)	W = 307, *p* = 0.04
Positive Aβ 42/40 ratio, N (%)	19 (59%)	5 (18%)	χ²(1, 57) = 13.491, *p* = 0.001
CSF p-Tau 181, pg/mL	53.0 (57.5)	43.8 (19.9)	W = 551, *p* = 0.128
Positive p-tau 181 biomarker, N (%)	16 (48%)	5 (18%)	χ²(1, 57) = 11.398, *p* = 0.003

Median and IQR are reported in the table if not specified (otherwise count and %). Sex differences were evaluated with the chi^2^ test, and all other comparisons were performed with Wilcoxon rank sum tests. The difference in positive AD biomarkers was tested with a logistic binary regression including age as a covariate. CSF AD biomarkers were adjusted for age. Measures of cognitive performance were adjusted for education and age. The adjustments were performed by regressing out the effect of the confounder before analysis. 60 participants had undergone CSF testing (32 LB and 28 HC). Cut off for CSF biomarkers were based on the unadjusted values. Cut-offs used were previously validated, including 0.062 for Aβ 42/40 ratio and 63 pg/mL for p-tau^52^.

*Aβ* amyloid beta, *AD* Alzheimer’s disease, *CSF* Cerebrospinal fluid, *FCSRT* Free and Cued Selective Reminding Test, *HC* Healthy controls, *LB* Lewy body, *MMSE* Mini Mental State Examination, *P-Tau* phosphorylated-Tau, *WAIS* Wechsler Adult Intelligence Scale, *WMSA* White matter signal abnormalities.

### Global WMSA

The patients with LB group had a significantly higher global WMSA volume than the HC group, after adjusting for age and estimated total intercranial volume (eTIV) (Table 1).

### Total and cholinergic pathways WMSA

The patients with LB had a higher absolute volume of WMSA in the whole cholinergic system compared to the HC group. The patients with LB group also had a higher volume of WMSA in cingulate and the external capsule pathways compared to the HC group (Table 2).

**Table 2:** MRI measures

	LB group	HC group	W-value/ χ²-value, p-value
Global WMSA, log_10_	3.46 (0.32)	3.26 (0.27)	*W* = 897, *p* = < 0.001
NbM volume, mm^3^	220 (77)	242 (60)	*W* = 377, *p* = 0.009
*Absolute cholinergic WMSA*			
Total cholinergic WMSA, log_10_	3.02 (0.40)	2.76 (0.29)	*W* = 971, *p* < 0.001
Cholinergic WMSA in cingulate pathway, log_10_	2.38 (0.49)	1.79 (0.62)	*W* = 970, *p* < 0.001
Cholinergic WMSA in External capsule, log_10_	3.02 (0.40)	2.76 (0.29)	*W* = 970, *p* < 0.001
Cholinergic WMSA in the frontal lobe, log_10_	1.51 (1.00)	0.57 (0.76)	*W* = 872, *p* < 0.001
Cholinergic WMSA in the temporal lobe, log_10_	1.17 (0.65)	0.87 (0.60)	*W* = 892, *p* < 0.001
Cholinergic WMSA in the parietal lobe, log_10_	2.09 (0.75)	1.68 (0.62)	*W* = 863, *p* = 0.001
Cholinergic WMSA in the occipital lobe, log_10_	1.97 (0.43)	1.83 (0.40)	*W* = 696, *p* = 0.222
Cholinergic WMSA in insula, log_10_	0.90 (0.72)	0.34 (0.69)	*W* = 865, *p* = 0.001
*Proportion Cholinergic WMSA*			
Proportion total cholinergic WMSA	0.37 (0.09)	0.31 (0.13)	*W* = 817, *p* = 0.008
Proportion cholinergic WMSA in cingulate pathway	0.09 (0.06)	0.04 (0.05)	*W* = 896, *p* < 0.001
Proportion cholinergic WMSA in external capsule pathway	0.37 (0.10)	0.31 (0.13)	*W* = 817, *p* = 0.008
Proportion cholinergic WMSA in Frontal lobe	0.14 (0.37)	0.06 (0.15)	*W* = 803, p = .012
Proportion cholinergic WMSA in Temporal lobe	0.88 (0.30)	0.75 (0.39)	*W* = 649, *p* = 0.513
Proportion cholinergic WMSA in Parietal lobe	0.77 (0.31)	0.82 (0.34)	*W* = 599, *p* = 0.957
Proportion cholinergic WMSA in Occipital lobe	0.79 (0.37)	0.80 (0.30)	*W* = 478, *p* = 0.165
Proportion cholinergic WMSA in Insula	0.57 (0.53)	0.01 (0.79)	*W* = 715, *p* = 0.149
*Mean diffusivity*			
MD in Cingulate pathway	9.97 × 10^−4^ (1.08 × 10^−4^)	9.40 × 10^−4^ (6.27 × 10^−5^)	*W* = 887, *p* < 0.001
MD in External capsule pathway	1.18 × 10^−3^ (1.21 × 10^−4^)	(1.12 × 10^−3^) (9.25 × 10^−5^)	*W* = 895, *p* < 0.001
MD in the non-cholinergic WM	8.38 × 10^−4^ (5.46 × 10^−^^5^)	8.12 × 10^−4^ (2.09 × 10^−^^5^)	*W* = 864, *p* = 0.001

Median and IQR are reported in the table. Global WMSA and the absolute measures of WMSA in the cholinergic system were log10 transformed. Global WMSA, NbM volume and the absolute cholinergic WMSA measures were adjusted for age and eTIV. For “proportion total cholinergic WMSA”, “proportion cholinergic WMSA in the cingulate pathway”, and “proportion cholinergic WMSA in the external capsule pathway”, the raw cholinergic WMSA measures were divided by global WMSA. For the four lobar proportion cholinergic WMSA measures and insula WMSA, raw cholinergic WMSA measures were divided by each lobe or insula WMSA. Proportional cholinergic measures and MD in the white matter pathways were adjusted for age.

*HC* Healthy controls, *LB* Lewy body, *MD* Mean diffusivity, *NbM* Nucleus basalis of Meynert, *WM* white matter, *WMSA* White matter signal abnormalities.

Since our aim was to elucidate whether there was a higher WMSA volume in the cholinergic system beyond and above the higher global WMSA volume in LB, we adjusted all cholinergic WMSA measures. Specifically, for the “total cholinergic WMSA”, we divided the total cholinergic WMSA by the global WMSA volume (called “proportion total cholinergic WMSA” from here and onwards). Similarly, we calculated the “proportion cholinergic WMSA in the cingulate pathway” and “proportion cholinergic WMSA in the external capsule pathway” by dividing WMSA in each pathway by the global WMSA volume.

Our findings showed that the patients with LB group had a higher proportion of total cholinergic WMSA compared to the HC group. The patients with LB group also had a higher proportion of cholinergic WMSA in the cingulate and in external capsule pathways compared to the HC group (Table 2).

### Cholinergic WMSA within lobes and insula

When absolute cholinergic WMSA was divided into the lobar regions and the insula, the patients with LB had a higher volume of cholinergic WMSA in the frontal, temporal, and parietal lobes and in insula compared to the HC group. (Table 2).

Since our aim was to investigate if there was a higher cholinergic WMSA in the lobes and insula beyond and above the higher global WMSA in LB, we adjusted the measures by dividing the volume of cholinergic WMSA in frontal, temporal, parietal, and occipital lobe as well as insula by the WMSA volume in each respective lobe or insula. WMSA volume in each respective lobe or insula was chosen instead for the global WMSA volume as the regional distribution of WMSA differs between patients with LB and controls^30^. To avoid division by zero, in cases with no WMSA in a particular lobe or insula, the adjustment was set to zero as there was no cholinergic WMSA (or any WMSA in the area).

Our findings showed that the patients with LB group had a higher proportion of frontal cholinergic WMSA compared to the HC group. We observed no statistically significant differences in the proportion of cholinergic WMSA in occipital, parietal, and temporal lobes or insula (Table 2). However, 85% (*n* = 28) of patients with LB had cholinergic WMSA in the insula, which was significantly higher than the frequency observed in the HC group (45%, *n* = 16, χ² (1, 69) = 10.479, *p* < 0.001).

### The association of WMSA with NbM volume and MD in cholinergic pathways

**Fig. 1:**
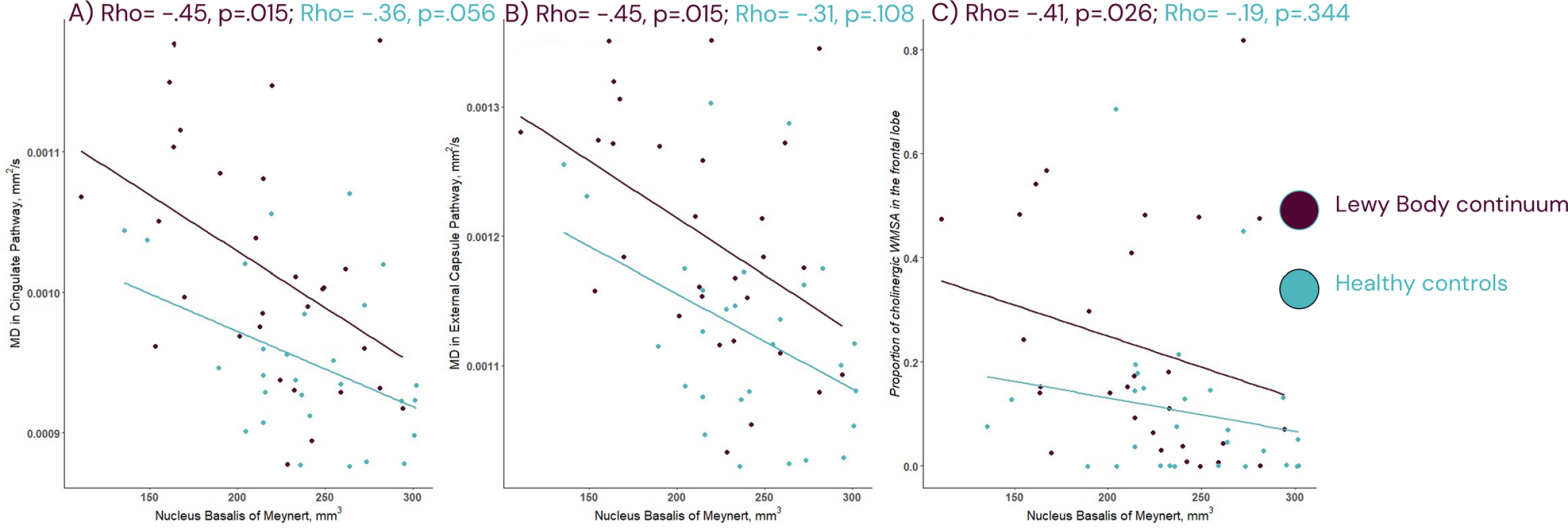
Associations with Nucleus basalis of Meynert (NbM) volume among patients on the Lewy body continuum and the healthy controls. All analysis are Spearman correlations. In **A** we display the association between mean diffusivity (MD) of the cingulate pathway and the volume of the NbM, **B** display the association between the MD of the external capsule pathway and the volume of the NbM, **C** display the association between the proportion of cholinergic WMSA in the frontal lobe with the volume of the NbM. Prior to analysis, NbM volume was adjusted for age and estimated intercranial volume, while MD in the cingulate and external capsule pathways as well as proportion of cholinergic WMSA in the frontal lobe were adjusted for age. WMSA, white matter signal abnormalities.

In line with our second aim of clarifying the impact of WMSA on biomarkers of neurodegeneration, we investigated NbM volume and MD in cholinergic pathways. The patients with LB group had a lower NbM volume and a higher MD in cingulate and external capsule pathways compared to the HC group (Table 1). In the patients with LB, a lower NbM volume correlated with a higher MD in both the cingulate and external capsule pathways, while these correlations were not statistically significant in the HC group (Fig. 1A, B).

Regarding the correlations between WMSA and NbM, a higher proportion of cholinergic WMSA in frontal lobe was associated with a lower NbM volume in the patients with LB. This association was not statistically significant in the HC group (Fig. 1C). There was no association for the proportion of cholinergic WMSA in other lobes or insula with NbM volume.

**Fig. 2:**
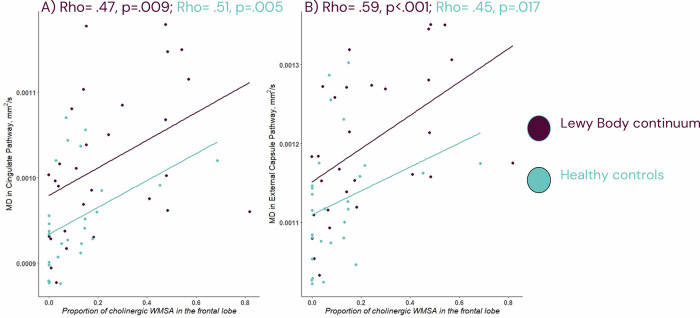
Associations between integrity in the cholinergic pathways and proportion of frontal cholinergic WMSA volume among patients on the Lewy body continuum and the healthy controls. All analysis are Spearman correlations. In **A** we display the association between mean diffusivity (MD) of the cingulate pathway and the proportion of cholinergic WMSA in the frontal lobe, in **B** we display the association between the MD of the external capsule pathway and the proportion of cholinergic WMSA in the frontal lobe. Prior to analysis, MD in the cingulate and external capsule pathways as well as proportion of cholinergic WMSA in the frontal lobe were adjusted for age. WMSA white matter signal abnormalities.

In terms of the association between WMSA and MD in cholinergic pathways, a higher proportion of frontal cholinergic WMSA was associated with a higher MD in cingulate and external capsule pathways in both patients with LB and HC (Fig. 2A, B).

### The association of WMSA with cognitive performance

**Fig. 3:**
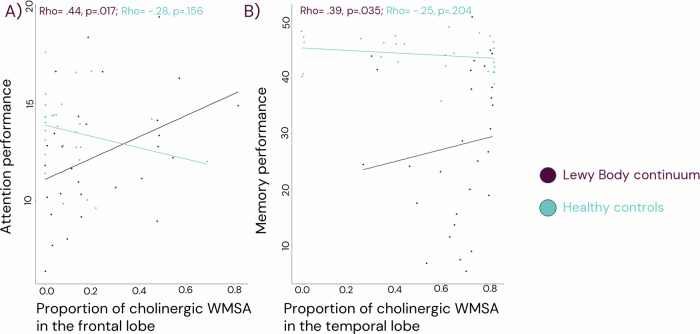
Associations between regional cholinergic WMSA and cognition among patients on the Lewy body continuum and the healthy controls. All analysis are Spearman correlations. In **A** we display the association between performance on an attention test (WAIS-III Digit span, total score) and the proportion of cholinergic WMSA in the frontal lobe. In **B** we display the association between the performance on a memory test (FCSRT free and cued recall, total score) and the proportion of cholinergic WMSA in the temporal lobe. Prior to analysis, the cognitive tests were adjusted for age and education while the proportion of cholinergic WMSA in the frontal lobe and temporal lobes were adjusted for age. WMSA white matter signal abnormalities.

We performed correlations for the global WMSA and all proportional cholinergic WMSA measures with the three included cognitive tests. In the patients with LB, a higher proportion of cholinergic WMSA in the frontal lobe was associated with a better performance on the attention test, and a higher proportion of cholinergic WMSA in the temporal lobe was associated with a better performance on episodic memory. Neither of these associations was significant in HC (Fig. 3A, B). Since these associations were in the opposite direction than what expected, to clarify the results we performed post hoc analysis for the correlation between the proportion of non-cholinergic WMSA in the frontal lobe and the attention test as well as for the proportion of non-cholinergic WMSA in the temporal lobe and episodic memory in the patients with LB. The proportion of non-cholinergic WMSA was calculated following the same formula as for the cholinergic WMSA described in the methods. We observed that a higher frontal non-cholinergic WMSA proportion was associated with worse performance on the attention test (Rho = −0.41, *p* = 0.023). Similarly, we observed a trend towards a higher proportion of non-cholinergic WMSA in the temporal lobe to be associated with worse episodic memory performance (Rho = −0.36, *p* = 0.052). We also followed these analyses with a correlation between the absolute values of cholinergic WMSA in the frontal lobe and the attention test (Rho = −0.18, *p* = 0.32), as well as the correlation between the absolute values of cholinergic WMSA in the temporal lobe and the episodic memory test (Rho = −0.21, *p* = 0.27).

There were no statistically significant associations other than the ones reported above.

### The association of WMSA with CSF biomarkers

A total of 62 participants had undergone CSF testing (32 patients with LB and 28 HC). As expected, the patients with LB had a higher proportion of Aβ positive cases and a lower Aβ 42/40 ratio than HC. The patients with LB also had a higher proportion of p-tau positive cases, whilst the difference in the continuous p-tau levels did not reach statistical significance (Table 1). In patients with LB, there were no statistically significant correlations between the proportional WMSA measures and CSF biomarkers.

## Discussion

Our overall goal was to investigate regional WMSA placement within cholinergic white matter pathways along the Lewy body (LB) continuum, spanning from MCI-LB to DLB. Our first aim was to characterise the placement of WMSA and evaluate if patients with LB are more likely to have WMSA in cholinergic pathways compared to controls. Our second aim was to clarify the impact of WMSA on cholinergic integrity and their associations with biomarkers of neurodegeneration and AD co-pathology as well as measures of cognition. We demonstrated that patients with LB have a higher proportion of WMSA in cholinergic pathways compared to controls. Specifically, the patients with LB had a higher proportion of cholinergic WMSA in the frontal lobe compared to the controls. In the insula, we did not observe any statistically significant difference in the proportion of cholinergic WMSA, but the patients with LB group more often had any cholinergic WMSA in the insula compared to the controls. In addition, we observed an association of the proportion of cholinergic WMSA in the frontal lobe with the integrity of cholinergic white matter pathways and NbM volume. Finally, we observed an association of the proportion of cholinergic WMSA in frontal and temporal lobes with cognitive performance in patients with LB.

In this study, we demonstrated that patients with LB are more likely to have WMSA in cholinergic pathways compared to healthy controls. We observed this both in absolute and proportion measures of cholinergic WMSA, i.e., when global WMSA volume is accounted for. This finding included both the cingulum and external capsule pathways as well as the total cholinergic WMSA. A higher volume of cholinergic WMSA in patients with neurodegenerative diseases has been reported in two previous studies using radiological visual ratings. Park et al.^6^ reported that a combined group of patients with DLB, AD, or PDD had more cholinergic WMSA than controls. In Jerele et al.^22^, we reported that patients with LB had more cholinergic WMSA compared to controls, which correlated with global WMSA. Unlike these previous studies, our present study is the first using an automated method for cholinergic WMSA^22^. Since automated methods are more sensitive than radiological visual ratings, our current approach helped us capturing a higher WMSA volume in the cingulate pathway in the patients with LB compared to controls, which we did not observe using the radiological visual rating in the same cohort in Jerele et al.^22^ Another novelty of our study is that we do not only investigate absolute volume as in the two previous studies, but we also investigate the proportion of cholinergic WMSA. This allowed us to assess whether cholinergic system is more frequently affected by WMSA than non-cholinergic WMSA as well as in comparison to controls. The proportion approach is important because patients with LB often have more WMSA than controls^27,28^, meaning that more cholinergic WMSA could just be explained by the overall higher WMSA volume in LB. We demonstrated that patients with LB are more likely to have a higher proportion of WMSA in cholinergic pathways compared to controls, irrespectively of their higher global WMSA. This finding may indicate a higher vulnerability to WMSA in the cholinergic system in patients with LB.

Our first aim was to not only evaluate WMSA in the cholinergic pathways but also to investigate their placement in brain lobes and insula. As such, we investigated the absolute volume of cholinergic WMSA in each lobe and insula, but also more importantly the proportion of cholinergic WMSA. We demonstrated that the patients with LB had a higher proportion of WMSA in the cholinergic white matter of the frontal lobe compared to the controls. Cholinergic white matter projecting through either the cingulum or the external capsule traverse areas associated with the frontal lobe^19^, for example in parts of the centrum semiovale or corona radiata. Sarro et al.^30^ reported widespread areas where patients with DLB had more WMSA than controls including regions in the frontal lobe. In contrast, we observed no statistical difference between the patients with LB and HC-groups in the proportion of WMSA in the cholinergic white matter of temporal, parietal, and occipital lobes and insula. Similarly, we observed no statistical difference between groups in the absolute cholinergic WMSA in the lobes and insula. However, we qualitatively observed that cholinergic WMSA in insula was not as common in HC while it was frequent in LB. Hence, we followed our main analysis to statistically investigate the presence of cholinergic WMSA in insula. This analysis demonstrated that the patients with LB group (85%) was statistically significantly more likely to have cholinergic WMSA in the insula compared to the HC group (45%). It has been previously reported that patients with LB have lower grey matter volume in the insula compared to controls, as well as that a higher WMSA volume is associated with a lower grey matter volume in the insula in DLB^31,32^. The neurodegenerative process of the insula may start in prodromal-DLB^31^ and progress over time, extending to other cortical areas that receive cholinergic input in the transition to DLB^33^. A previous study showed that the insula is affected not only by α-synuclein but also Aβ and p-tau-related pathologies^34^. Our current findings suggest that WMSA may be an additional contributor to the neurodegeneration of the insula in patients with LB, at least for the cholinergic white matter adjacent to the insula.

Our second aim was to clarify the impact of WMSA on cholinergic integrity and their associations with biomarkers of neurodegeneration and AD co-pathology. We did not observe any statistically significant association of regional cholinergic WMSA with CSF biomarkers of AD co-pathology in our analyses. This was partially expected as there were no associations in Jerele et al.^22^, when this association was evaluated with global WMSA measures. Hence, our current data contributes to the conclusion that AD-related pathology may not be one of main contributors to the neurodegeneration of cholinergic pathways in aging, AD or LB^20,22,25^. Instead, we found significant associations of cholinergic WMSA with NbM volume and integrity of cholinergic pathways. We demonstrated that a higher proportion of frontal cholinergic WMSA was associated with lower NbM volume in the patients with LB group, but not in the HC group. Involvement of the NbM is well documented in patients with LB^13,14,35^. The observed associations could aid in understanding the contribution of WMSA towards cholinergic dysfunction in LB. This is further supported by our study demonstrating an association between cholinergic WMSA in the frontal lobe and the integrity of the cholinergic pathways in both the LB and the HC groups. Previous studies showed that a higher global WMSA volume is associated with decreased thickness or volume in grey matter in areas receiving cholinergic innervation, especially in frontal areas in DLB but also in the insula^32,36^. Further, disruption of the white matter connectivity is pronounced in DLB and can proceed MRI measures of grey matter neurodegeneration^37^. We have earlier suggested that decreased integrity in the cholinergic system may be an earlier marker of cholinergic degeneration than NbM volume in the AD continuum^25^.

Finally, we aimed to clarify the impact of cholinergic WMSA on cognitive measures. In our recent publication by Jerele and collegues^22^, we did not observe any significant associations between global measurements of WMSA with cognitive measures in the same cohort that used in the current study. Generally, the role of WMSA on cognition in DLB is unclear with some publications suggesting an effect on global cognition and other not^27,28,38^. However, as cognitive impairment in specific domains correlates with alterations in specific brain networks or regions, in the current study we hypothesised that using our regional and proportional method to evaluate cholinergic WMSA in different lobes of the brain and insula would capture associations with cognitive measures. We selected measures of global cognition as well as attention and memory, because previous studies showed their association with cholinergic white matter pathways^11,21,25^. We observed in our analysis that patients with LB with a higher proportion of cholinergic WMSA had better cognitive performance, which was not observed among the controls. Specifically, a higher proportion of frontal cholinergic WMSA was associated with a better performance in attention, and a higher proportion of cholinergic WMSA in the temporal lobe was associated with a better performance in episodic memory. There are some previous publications with a regional approach to WMSA and cognition. Membreno and collegues^39^ reported an association between higher temporal WMSA and worse memory in cognitively intact individuals and people with MCI^39^. Pozorski and collegues^40^ reported an association between more WMSA primarily in the frontal lobe with worse memory as well as an association between higher WMSA in frontal, temporal, and parietal lobes with worse executive function in patients with Parkinsons disease (PD)^40^. Coenen et al.^41^ showed that higher total WMSA but also more WMSA in the left and right anterior thalamic radiation and forceps major were associated with worse memory performance. Likewise, more WMSA in the left inferior fronto-occipital fasciculus, anterior thalamic radiation, and forceps major were associated with lower performance of attention and executive ability in participants ranging from subjective cognitive complaints through to dementia^41^. The anterior thalamic radiation traverses the frontal lobe and the inferior fronto-occipital fasciculus traverses both the temporal lobe and the frontal lobe, with partial overlap with cholinergic white matter. As it can be noted, all these previous studies showed that a higher WMSA volume correlated with worse cognitive performance, while in our study we observed that a higher proportion of cholinergic WMSA correlated with better cognitive performance. One interpretation of our result is that a higher proportion of WMSA in the cholinergic white matter reflects a lower proportion of WMSA in the non-cholinergic white matter. We therefore performed post-hoc analysis where we evaluated the association between the proportion of non-cholinergic WMSA in the temporal lobe with episodic memory and proportion of non-cholinergic WMSA in the frontal lobe with attention. Our results showed that, indeed, a higher proportion of non-cholinergic WMSA was associated with worse cognitive performance. Thus, a higher proportion of WMSA in the cholinergic system is not protective but rather reflects that there is less WMSA placed in strategically more detrimental locations for the selected cognitive tests in LB. This interpretation is also supported by our finding of no statistically significant associations between the absolute values of cholinergic WMSA in the frontal or temporal lobes and the cognitive tests, as the absolute value unlike the proportion does not depend on the proportion of WMSA outside of the cholinergic system.

This study has some limitations. Our patients with LB were older and had lower education than the HC group. To circumvent this, we adjusted all our variables for age before statistical analysis and additionally for education when investigating cognitive measures. The adjustments ought to have limited the effects of age and education, but our findings need to be interpreted accordingly. All our participants had a clinical diagnosis without postmortem confirmation. However, there is a reasonable correspondence between clinical and postmortem diagnosis as shown in previous research^42^. The proportion of cholinergic WMSA is an approach that has not been used in previous studies and the possibility of a selective vulnerability in the cholinergic system needs to be further evaluated in other cohorts. We used the WAIS-III Digit span as a measure of attention^43,44^. However, we acknowledge that the Digit span is also a test of working memory^45^, which may make Digit span additionally tap on non-cholinergic frontal white matter. An advantage of Digit span is that it does not contain any visuospatial component, hence avoiding the influence of visuospatial impairment commonly reported in DLB and prodromal DLB^4,5^. Finally, whilst our sample size is in-line with previous publications in DLB focussing on diffusion MRI, statistical power may have been insufficient to detect more discrete associations.

To conclude, patients on the LB continuum have more WMSA in cholinergic system compared to controls, both in terms of absolute volume and as a proportion of WMSA above and beyond their higher global WMSA volume. Specifically, patients with LB had a higher proportion of cholinergic WMSA in the frontal lobes, which was associated with neurodegeneration in the NbM and cholinergic white matter pathways. We also demonstrated associations of cholinergic WMSA in frontal and temporal lobes with attention and memory cognitive tests, respectively. Our data thus suggest that there may be a selective vulnerability to WMSA in the cholinergic system of patients with LB. These findings also highlight the importance of a regional approach to understanding the role of WMSA. Further endeavours should strive for a greater understanding of the longitudinal changes of WMSA and cholinergic degeneration in individuals with LB. Our current findings contribute to the consolidation of the recent evidence on the interplay between WMSA and cholinergic neurodegeneration in the Lewy body continuum^11,12,22,36^.

## Methods

### Participants

We included a total of 71 participants (33 patients with LB and 36 cognitively unimpaired controls) from the Sant Pau Initiative on Neurodegeneration (SPIN) cohort^46^. Patients with LB were recruited at the Sant Pau Memory Unit (Barcelona, Spain) between June 2013 and December 2017. The patients with LB were diagnosed following the diagnostic criteria for probable DLB^4^ or MCI-LB^5^. Specifically, 12 had a diagnosis of probable DLB and 21 had a diagnosis of MCI-LB at the time of MRI acquisition. The diagnosis was determined by neurologists through extensive neurological and neuropsychological evaluations in addition to clinical MRI scans and cerebrospinal fluid (CSF) measures. The 36 controls had no cognitive complaints, no impairment in daily living and, per selection, their Mini-Mental State Examination (MMSE)^47,48^ total score was ≥27. Further details about the selection criteria in the SPIN cohort are available elsewhere^46^.

In this study, MMSE was used as a measure of global cognition. Since the cholinergic system is primarily involved in attention and episodic memory^49^, we included the Free and Cued Selective Reminding Test (FCSRT) free and cued recall total score as a measure of episodic memory^50,51^ and the WAIS-III Direct and Reverse Digit span total score as a measure of attention^43,44^.

CSF assessment is described in detail elsewhere^46^. Briefly, CSF samples were analysed for the Aβ 42/40 ratio and phosphorylated-Tau at threonine 181 (p-tau) using an ELISA. Cut-offs used were previously validated, including 0.062 for the Aβ 42/40 ratio and 63 pg/mL for p-tau^52^.

All procedures in this study were approved by the local Ethics Committee in Barcelona, Spain following the Helsinki Declaration. All the participants supplied informed written consent.

### MRI scanning

All participants were scanned with the same 3 T Philips Achieva scanner (Philips Healthcare) following the SPIN protocol^46^. Two MRI sequences were used in our analyses: a high-resolution 3D T1-weighted magnetisation prepared rapid gradient echo (MPRAGE) sequence, with 8.1 ms repetition time, 3.7 ms echo time, 160 slices with a slice thickness of 1 mm and a voxel size of 0.94 × 0.94 × 0.94 mm; and a diffusion-weighted echo-planar sequence with a 10,800 ms repetition time, 78 ms echo time, 80 slices with a slice thickness of 2 mm, a voxel size of 2 × 2 × 2 mm, 32 isotropically distributed gradient orientations (*b* = 1000 s/mm^2^), along with a single *b* = 0 image and an additional *b* = 0 image with inverted phase encoding. The data, pre-processing and storage were managed in the HiveDB^53^.

### Segmentation of NbM and cholinergic tractography

We segmented the NbM using a region of interest created from postmortem data^54^. The subsequent tractography method is described in detail elsewhere^21,25^. Briefly, the diffusion-weighted MRI data was pre-processed using FSL (FMRIB Software Library)^55^. Probabilistic tracking was performed through the repetition of 5000 random samples from each voxel within the NbM mask^56^. To be retained, the generated streamlines had to pass through either mid-way cingulum or external capsule masks from the Johns Hopkins white matter atlas^57^, available in FSL. We also applied exclusion masks for the brainstem (from FSL’s First segmentation routine)^58^ and the anterior commissure^21^. In the next step, we created an unbiased template by using the pre-processed b0 images. Both pathways (through the cingulum and external capsule) from all HC cases were then non-linearly warped into the space of this unbiased template. Finally, pathway-specific binary masks were created by retaining voxels present in at least 60% of the individual warped tracts Next, we calculated the index of mean diffusivity (MD) to evaluate the microstructure properties of the two resulting cholinergic pathways for each participant, as in previous publications^11,12,20–22,25^.

### Segmentation measures of WMSA

The focus of this study was WMSA in the cholinergic system. To approach our aim, we estimated seven different WMSA measures spanning from more global to lobar measures of WMSA. We started with estimating the “global WMSA volume”, which is an estimation of all WMSA in the brain. We did this by performing automated segmentation of WMSA volume on T1-weighted images with the FreeSurfer v7.3.1 Image Analysis software^59^. Specifically, FreeSurfer relies on an algorithm that labels each voxel based on local and intensity-related probabilistic information based upon estimations from a built-in training dataset^59^. WMSA from T1 correlate strongly with WMSA from fluid-attenuated inversion recovery MRI^20,60–62^.

Next, we continued with several estimations of cholinergic WMSA. We calculated the total cholinergic WMSA volume, which is an estimation of all WMSA overlapping with the white matter of cholinergic pathways. We also calculated two measures of WMSA specifically in the cingulum and the external capsule pathways of the cholinergic system. To estimate these three measures, we overlapped the global WMSA segmentation with the tract-specific binary masks for the cingulum and external capsule described in the previous section. This gave us estimates of “cholinergic WMSA in the cingulate pathway” and “cholinergic WMSA in external capsule pathway”. Afterwards, we combined these two pathway measures to create an estimate of the “total cholinergic WMSA” volume.

Additionally, we estimated the cholinergic WMSA in each brain lobe. To do that, we first segmented the white matter with labels of the adjacent cortex extended 5 mm inwards^63^. Then, we combined all these segmentations into four lobes and insula as described in Klein & Tourville^64^. We overlapped the “global WMSA” and the “total cholinergic WMSA” with these segmentations of lobes and insula, which provided us with measures for total WMSA and cholinergic WMSA in each area (i.e. cholinergic WMSA in frontal, temporal, parietal, and occipital lobe as well as cholinergic WMSA in insula). The calculation of cholinergic WMSA allowed us to also calculate non-cholinergic WMSA by estimating WMSA outside of the cholinergic masks.

For all these WMSA measures but the global WMSA volume, we estimated both absolute and proportional measures as further elaborated in the results section. Absolute measures of WMSA were adjusted for eTIV and age (see below). The eTIV was calculated through FreeSurfer v7.3.1 Image Analysis software^59^.

### Statistical analysis

Our aim was to evaluate the differences between the LB and HC groups in terms of WMSA in the cholinergic system and evaluate differential associations of WMSA with neurodegeneration, cognition, and CSF biomarkers within each group (e.g. the association of cholinergic WMSA and NbM volume separately in patients with LB and HC groups). Due to WMSA measures not being normally distributed, we transformed absolute volumetric measures of cholinergic WMSA with log10 transformation. In the regions that contained cases with no cholinergic WMSA, all participants had their volume increased by 1 to facilitate the transformation. While log10 transformation approximated WMSA measures towards the normal distribution, main statistical analyses were performed using robust statistical tests against slightly skewed distributions. Specifically, group differences between patients with LB and HC were assessed with the Wilcoxon rank sum test for continuous variables and the Chi-square test or logistic binary regression for dichotomous variables; and Spearman’s correlations were used to test for associations between pairs of continuous variables.

Furthermore, we adjusted the study variables to remove the influence of confounders before statistical analysis. More specifically, we regressed out the effect of age from all measures except education and sex with a linear regression model. For the volumetric measurements of global WMSA, cholinergic WMSA and NbM, we adjusted for eTIV in addition to age. Cognitive measures were adjusted for education in addition to age. All adjustments were performed with the formula proposed by Voevodskaya et al.^65^, by computing mean and beta values from multiple linear regression models solely in the HC group, where the variable of interest was the outcome variable and the confounder was predictor. For variables adjusted for more than one confounder, they were fitted in the same model. The adjustment was performed in both patients with LB and controls.

R Studio was used for all statistical analysis and α was set to 0.05 and confidence intervals to 95%.

## Data Availability

Data may be available upon reasonable request if legal and ethical requirements can be met.
